# A "Red Cross" Warning

**Published:** 1921-05-07

**Authors:** 


### A "Red Cross" Warning.

It is interesting to note that in a bulletin issued to the public, the League of Red Cross Societies refer to the connection between public health problems and the present widespread unemployment in almost the same terms used recently in our own columns. The alarming effect, says the League, which this unemployment has already had on the health of the public must necessarily arouse a feeling of great anxiety. Up to the middle of last year, despite the considerable increase in the cost of living, which had not been compensated by a proportional increase of wages, the health of the public had not suffered, as far as could be ascertained. This was largely due to the absence of unemployment, the regular earning of wages balancing in a certain measure the disproportion between salaries and the cost of living.

For some months past the situation has been entirely changed, unemployment is spreading continually, and already its influence on the labouring class is noticeable. Against its evil effects only a powerful, well-organised, and well-directed force can be of any avail, especially as concerns bringing relief to thousands of families which have fallen on evil days. The problem is a complex one, of great urgency, and very serious. It can be said that there exists at present no institution, whether Governmental or private, which is capable of taking in hand and resolving the grave economic and public health problems resulting from unemployment. The Red Cross alone, thanks to the powerful means at its disposal, to the extension of its spheres of action and to the moral support of public opinion, could be qualified to undertake this difficult task. The first question is to intensify the relief work of all existing public health institutions, and especially of those concerned with the fight against tuberculosis. Another important question is to find a satisfactory solution for the grave housing problem. It is the duty of the Red Cross to make every effort to enable the working class to retain the improved sanitary conditions which it had been able to realise in recent times. The present situation might also serve as an occasion for organising in a permanent manner the fight against the sufferings and hardships due to unemployment, not only as concerns the present, but also with regard to the future.

